# Implementation of Surfactant Administration through Laryngeal or Supraglottic Airways (SALSA): A Jordanian NICU’s Journey to Improve Surfactant Administration

**DOI:** 10.3390/children9081147

**Published:** 2022-07-30

**Authors:** Naser Aldain A. Abu Leyah, Abeer A. Hasan, John N. Juneau, Maryam Ali Al Jammal, Ghada A. Jaber, Gregory E. Wilding, Kari D. Roberts, Scott O. Guthrie

**Affiliations:** 1 Department of Pediatrics, Division of Neonatology, Maternity and Children’s Hospital at Al Bashir Hospital, Amman 11151, Jordan; abeerjammaldr@yahoo.com (A.A.H.); aljammal.maryam96@gmail.com (M.A.A.J.); ahmadghada88@gmail.com (G.A.J.); 2 Department of Pediatrics, Division of Neonatology, University of Louisville, Louisville, KY 40202, USA; john.juneau@louisville.edu; 3 Department of Biostatistics, State University of New York, Buffalo, NY 14214, USA; gwilding@buffalo.edu; 4 Department of Pediatrics, Division of Neonatology, University of Minnesota, Minneapolis, MN 55454, USA; rober694@umn.edu; 5 Department of Pediatrics, Division of Neonatology, Vanderbilt University School of Medicine, Nashville, TN 37232, USA; scott.o.guthrie@vumc.org

**Keywords:** Respiratory Distress Syndrome (RDS), surfactant, LMA, supraglottic airways (SALSA), intubation–surfactant–extubation (InSurE)

## Abstract

Administration of liquid surfactant through an endotracheal tube for the treatment of respiratory distress syndrome has been the standard of care for decades. Surfactant administration through laryngeal or supraglottic airways (SALSA) is a simplified procedure for delivery of surfactant that is less invasive and better tolerated. The Al Bashir Maternity and Children’s Hospital NICU in Amman, Jordan, implemented SALSA as a potentially better practice in 2019 with the objective to effectively and efficiently deliver surfactant in a minimally invasive way and to decrease the adverse events associated with intubation–surfactant–extubation (InSurE) and laryngoscopy. The quality improvement initiative was conducted from March 2019 to December 2019. All infants who weighed 750 g or more who required surfactant were eligible. As physicians were trained in the technique and use expanded, we were able to use plan–do–study–act cycles to observe differences between SALSA and InSurE. The primary aim was the optimization of non-invasive ventilation by the effective and efficient delivery of surfactant. Balancing measures included episodes of bradycardia while receiving surfactant or the need for a second dose of surfactant. We evaluated 220 infants who received surfactant by SALSA or InSurE with a mean gestational age of 32 weeks and a mean birth weight of 1.8 kg. The Respiratory Severity Score (RSS) prior to surfactant administration was 2.7 in the SALSA group compared to 2.9 in the InSurE group (*p* = 0.024). Those in the InSurE group had a lower mean heart rate during the procedure (*p* =< 0.0001) and were more likely to need a second dose of surfactant (*p* = 0.026) or require intubation for mechanical ventilation (*p* = 0.022). Both groups were effectively delivered surfactant as evidenced by improvement in their RSS over an 8 h period. SALSA was a more time efficient surfactant delivery method (93 vs. 111 secs, *p* =< 0.0001). Implementation of SALSA into the Al Bashir NICU was successful. We found that it was equally effective to InSurE, but was a more efficient method of delivery. Infants who received surfactant by this method tolerated it well.

## 1. Introduction

### 1.1. Problem Description

Respiratory Distress Syndrome (RDS) is one of the most frequently diagnosed con dit ions in the neonatal intensive care unit (NICU) [1]. It is primarily a disease of pre ma tur ity caused by pulmonary surfactant deficiency which leads to increased alveolar col lapse, ventilation –perfusion mismatching and, ultimately, hypoxemia in neonates [2,3]. 

RDS remains a major cause of increased morbidity and mortality in neonates worldwide and especially in the low- and middle-income countries (LMICs). In a study conducted in Pakistan in 1997, mortality rates from RDS were as high as 41% and equivalent to mortality rates prior to the routine use of surfactant [4]. Jordan has reported similar findings and, in 2019, Khader et al. reported that pre-term and low-birth-weight neonates were almost 20 times more likely to die during the neonatal period compared to full-term infants in Jordan, with RDS being the most likely cause of death [5]. 

Early administration of surfactant has greatly improved outcomes for neonates with RDS [6]. Surfactant is now readily available worldwide, but utilization can be limited by the availability of practitioners skilled in intubation, as the traditional method of admin istration requires placement of an endotracheal tube. In addition, providers trained in intubation may not have the opportunity to maintain the skill. Two studies have shown that although skills may be learned at one time, if not routinely used, the skills are lost with technical ability returning to pre-training levels [7,8]. 

In addition to intubation requiring a skilled provider, it is also associated with well-known complications. These may occur due to multiple attempts and/or prolonged duration of the procedure which may be needed for success. Complications can include oral or tracheal trauma, right main bronchus intubation, accidental extubation, hypox emia, bradycardia, and changes in systemic or pulmonary blood pressures [9,10,11]. 

With the associated risks of airway manipulation and placement of an ETT along with the trend of less invasive respiratory support techniques being utilized, multiple techniques have been developed to administer surfactant while minimizing side effects to the neonate [12]. These techniques include: intubation –surfactant–extubation (InSurE), less invasive surfactant administration (LISA), minimally invasive surfactant therapy (MIST), and surfactant administration through laryngeal or supraglottic airways (SALSA). 

The InSurE method is the most widely used method to administer surfactant. The goal is to place an ETT for the delivery of the surfactant followed by prompt extubation back to non-invasive respiratory support [13]. This method is aimed to avoid prolonged mechanical ventilation (MV) which has been associated with increased risk for lung injury and bronchopulmonary dysplasia (BPD) [14]. A limitation to this method is that it still requires intubation to administer the surfactant. In a systematic review of 1674 patients who underwent the InSurE method, one third (median of 33%) of infants failed and required MV [15].

Other techniques include methods that use thin catheters to deliver surfactant through the vocal cords, such as LISA and MIST. Although these methods are considered overall less traumatic to the airway, they still require a provider skilled in laryngoscopy to place the catheter through the vocal cords [16,17]. 

The most recent method developed to minimize the negative effects and risks of airway manipulation is the SALSA technique. [18] A supraglottic airway device (SAD) has traditionally been used to obtain an airway when intubation has been difficult or unsuccessful and has been shown to be equivalent to intubation in efficacy for providing positive pressure ventilation [19,20]. With the SALSA technique, an SAD is used to administer surfactant. In contrast to previously noted methods, since the device rests in the posterior pharynx, nothing is advanced through the vocal cords, therefore, use of a laryngoscope is not needed. Since laryngoscopy is not needed, provider skill required for proper placement is dramatically decreased which is a major advantage of this method [21]. In a previous study, placement of the device took an average of 88 s with 67% of attempts being successful in less than 35 s [22]. Additional studies have shown that the SALSA technique decreases the need for MV, thereby improving success on non-invasive modes of ventilation and decreasing the risk of ventilator induced lung injury [23,24,25,26]. The SALSA technique is ideal in a LMIC setting where providers skilled in intubation and mechanical ventilators may not be available. Thus, this technique has the potential to improve global use of surfactant and improve outcomes for neonates [27].

### 1.2. Setting and Rationale

The Hashemite Kingdom of Jordan is a country in the Levant. It is considered by the World Bank to be middle income [28]. Jordan is recognized as a hub of medical tourism in the Arab World due to the medical systems’ reputation and commitment to the application of evidence-based care.

Al Bashir Hospital is the largest governmental hospital system in Jordan. It is a tertiary hospital that receives referrals from most hospitals throughout the country. It is also a teaching center for multiple residency programs and three major universities. Five main hospitals make up the system and include: Al Bashir Internal Medicine Hospital, Al Bashir General and Special Surgery Hospital, Al Bashir Emergency and Trauma Hospital, Al Bashir Interventional Radiology and Hematology/Oncology Hospital and the Maternity and Children’s Hospital. On average, there are approximately 4200 patients seen at these facilities each day.

The Maternity and Children’s Hospital has a capacity of 450 beds. In 2019, there were 16,309 births and 4698 admissions to the NICU which maintained an average daily census of 100 patients in its 95 beds capable of supporting all levels of care. RDS is the most common diagnosis in the NICU and 828 babies were given surfactant in 2019 in accordance with a clinical pathway developed by the Jordanian Ministry of Health.

Prior to this project, surfactant had solely been administered through an ETT at the Al Bashir NICU. This can be a difficult skill for the resident physicians to master. Once mastered, it may then be a difficult skillset to maintain as a general pediatric practitioner may have few chances to intubate depending on their setting. Faculty were introduced to the SALSA technique during an annual neonatal conference in Amman in 2019 and learned the procedure during a workshop. It was recognized that this method of surfactant delivery was less invasive, easy to teach, easy to learn, the practitioner could immediately implement the technique into their practice, and that there would be minimal to no degradation of skills over time. It was further realized that SALSA could be a technique which would help newborns in Jordan easily receive the well-recognized advantages of surfactant replacement therapy regardless of the location of their care.

We sought to implement this practice into the Al Bashir NICU to improve the care of our patients and to educate trainees on a method that could be easily used in their future practice.

### 1.3. Aim

The SALSA technique was introduced as a less invasive method to deliver surfactant in the Al Bashir NICU with the goal to optimize success on non-invasive ventilation. The expectation was that SALSA would be an effective and efficient method to deliver surfactant in infants who traditionally received InSurE in the NICU. We also sought to decrease the adverse events associated with surfactant administration by laryngoscopy.

## 2. Materials and Methods

### 2.1. Implementation

**Figure 1:**
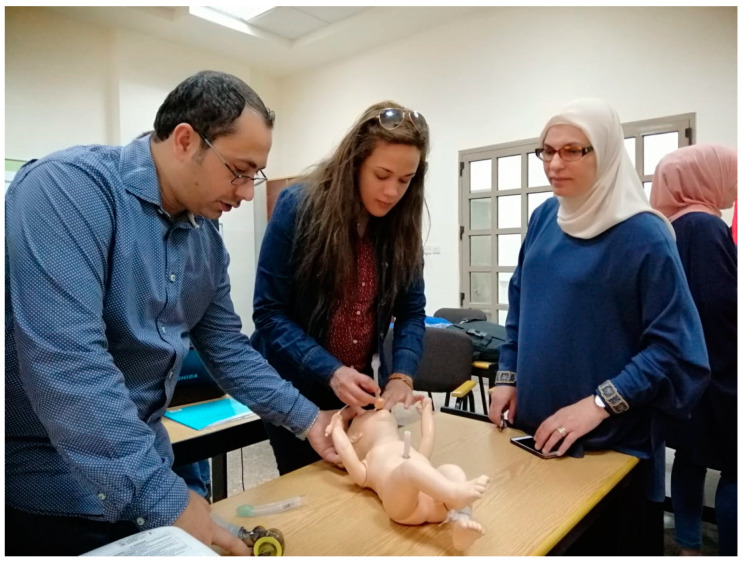
Al Bashir SALSA workshop used to educate providers on the technique.

Two physicians who had experience with the use of an SAD were identified as Master Trainers (MT) of the SALSA technique. Initial training for these providers was obtained during a workshop at the annual neonatal conference. The MT taught other providers in the unit the skills needed for the procedure and evidence-based practices on the proper clinical situation for use. This was done by taking already established neonatal resuscitation program workshops held periodically in the unit and adding an educational program on the use of an SAD in resuscitation and also for surfactant administration in the neonate. Educational presentations discussed device type and size, recommended use, procedural steps, and benefits over intubation. Learners were then able to practice placement of the SAD on mannequins with MT guidance. (Figure 1) Faculty and residents were encouraged to consider using the SALSA technique with the right clinical situation.

### 2.2. Intervention

The IRB of the Jordanian Ministry of Health reviewed and approved our quality improvement project. In accordance with Jordan’s clinical pathway for RDS management, all infants who receive surfactant must have clinical signs of RDS at less than 36 h of life and an FiO_2_ requirement of >40%. All neonates who weighed 750 g or more were a candidate as long as they were on non-invasive respiratory support prior to surfactant administration. Neonates with medical conditions such as congenital heart disease, metabolic disorders, genetic syndromes, neonatal encephalopathy, or who were requiring mechanical ventilation prior to surfactant administration were not considered optimal patients for SALSA and were excluded.

For SALSA, neonates received surfactant through an SAD (i-Gel, Intersurgical Ltd., Berkshire, UK, Size 1). A standard dose of 3 mL/kg of surfactant (Infasurf, ONY Biotech, Amherst, NY, USA) in two divided aliquots followed by brief PPV was then given. Cardiac monitoring was continuous during the procedure. Per workshop recommendations, atropine was given prior to placement of the SAD. Once surfactant was administered, the device was removed, and the infant was placed back non-invasive respiratory support with a standard PEEP of +5 cm H_2_O.

**Figure 2:**
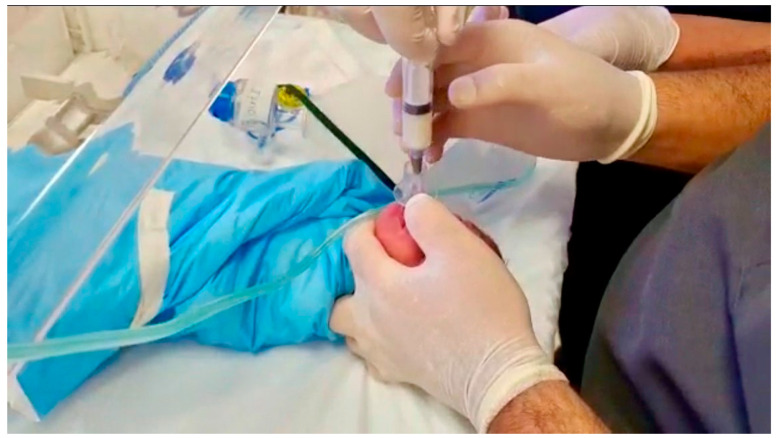
Master trainer using the SALSA technique to administer surfactant to a neonate.

A pilot test of SALSA was implemented into the NICU by the MT (Figure 2). When they were caring for a patient who would typically qualify for InSurE, they would implement SALSA. In this way, the staff caring for the patient could get used to the procedure, observe it in a clinical situation, and changes could be made to the plan if required as the technique continued to be implemented in the NICU.

As part of the plan–do–study–act cycles, other faculty and resident physicians were taught and then encouraged to use SALSA in similar clinical situations. The use of InSurE, however, was allowed to continue.

### 2.3. Study of the Intervention

As part of the evaluation of our quality improvement initiative to implement a less invasive method to deliver surfactant, we reviewed the charts of 220 neonates who had received surfactant in the Al Bashir NICU.

From March 2019 to December 2019, 110 infants had received surfactant by SALSA. Patients who had received SALSA were matched with contemporaneous patients with similar gestational age and weight who had received surfactant by InSurE.

### 2.4. Measures

Balancing measures collected included: change in oxygen saturations pre- and post-surfactant, change in oxygen requirement pre- and post-surfactant, respiratory severity score pre- and post-surfactant, whether a second dose of surfactant was required, complications such as a pneumothorax, the need for mechanical ventilation within 72 h of receiving surfactant, the lowest observed heart rate during the procedure, and duration of the procedure.

Process measures included physician and nursing education and structure measures included a written policy and availability of a SALSA flowchart to guide care.

As part of our aim, the expectation was that SALSA would be an effective and efficient delivery of surfactant. To evaluate this, we defined effective delivery as a subsequent decrease in the Respiratory Severity Score (RSS; positive end expiratory pressure (PEEP) x fraction of inspired oxygen (FiO_2_)) after receiving surfactant [29]. Efficient delivery was determined by the time it took to deliver surfactant. This was timed in seconds from the moment a laryngoscope or SAD entered the mouth until the time the ETT or SAD was removed.

### 2.5. Statistical Analysis

Descriptive statistics such as frequencies and relative frequencies were computed for all categorical variables. Numeric variables were summarized using simple descriptive statistics, such as the mean and standard deviation. Associations between group and categorical variables were assessed using standard contingency tables. In this case, independence was tested using Fisher’s exact test. Differences in groups with regards to numeric variables were examined through calculation of the arithmetic mean differences and statistically tested using Pitman’s permutation test. For RSS, which was measured longitudinally, within- and between-group differences were statistically assessed using a multivariate linear model. In taking this approach, RSS was fit as a function of group, time, and the interaction between groups with time. To account for the within-subject dependence structure, the model assumes that the distribution of the error terms for each subject to be multivariate normal with zero mean and a first-order autoregressive structure. Once a model was fit, specific linear contrasts based on the estimated model parameters were constructed and used to test hypotheses of interest. Standard diagnostic plots were used to assess model fit. A nominal significance level of 0.05 was used in testing and all analyses were carried out using SAS version 9.4 statistical software (Cary, NC, USA).

## 3. Results

The two study groups compared were similar in regards to sex, gestational age, and birth weight as seen in Table 1. There was a statistically significant difference in the RSS at time of surfactant administration, heart rate during the procedure, and receipt of atropine prior to any procedure. Table 2 demonstrates important clinical outcomes. Infants who received their first dose of surfactant by InSurE were significantly more likely to require a second dose of surfactant and be intubated for mechanical ventilation, and although non-significant, be treated for a pneumothorax in comparison to those who were treated by SALSA.

**Figure 3:**
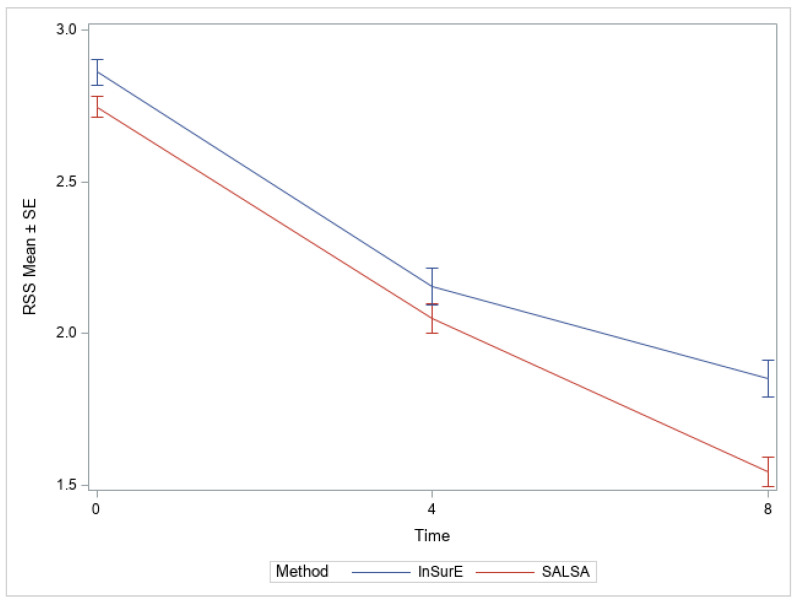
Respiratory Severity Score at time of surfactant administration and at intervals up to 8 h post-surfactant.

Figure 3 demonstrates the effect of surfactant on RSS by mode of surfactant delivery over an 8 h period. The two groups did have a statistically significant difference in RSS at the time of treatment (*p* = 0.024), but both methods of surfactant administration demonstrated the desired effect as noted in the drop of the RSS by 8 h after treatment which was statistically significant within both study groups (*p* < 0.0001). A significant interaction of method with time was found (*p* = 0.0002), with RSS group differences at 8 h being the largest across time and statistically significant (*p* = 0.0004).

**Figure 4:**
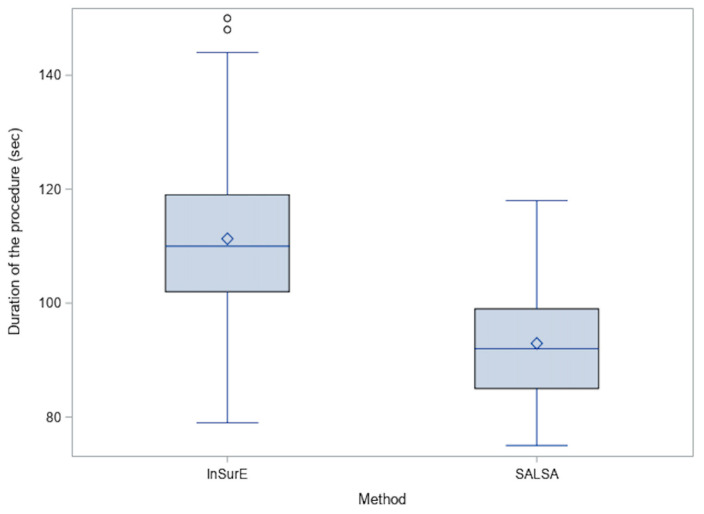
Duration of the procedure.

Figure 4 shows the time required to perform InSurE or SALSA. Administration by SALSA was more time efficient in comparison to the InSurE group (92.9 vs. 111.3 secs, *p* < 0.0001).

## 4. Discussion

### 4.1. Summary

The Al Bashir NICU was able to successfully implement SALSA as a minimally invasive form of surfactant administration. Our data would suggest that this is an effective form of surfactant delivery, is more efficient than InSurE, and is well tolerated by the patient.

### 4.2. Interpretation

SALSA is a simple procedure which effectively and efficiently delivers surfactant. It avoids the need for mastery of laryngoscopy and the maintenance of this skill. It allows any provider to place a device into the larynx and administer surfactant into the trachea to treat RDS in an infant who is maintained on non-invasive respiratory support. The ease of the SALSA technique, safety profile, and minimal equipment needed can allow it to be used in a variety of settings. Our hope in teaching this to our resident physicians is that they will use this technique in their practice and at the hospitals which typically refer patients to Al Bashir.

### 4.3. Limitations

We recognize that there are several limitations to our report. First, it is retrospective in nature. We attempted to overcome this by appropriate selection of case controls to evaluate SALSA as a potentially better practice in the NICU. Since the clinician was allowed to choose between SALSA or InSurE at the bedside, we found that selection bias was likely. Patients that appeared more ill (higher RSS at time of surfactant admin istration), seemed to have been more likely to be selected to receive InSurE. The InSurE group was more likely to require a second dose of surfactant and also more likely to require mechanical ventilation. This likely patient selection bias, however, does not detract from the finding that SALSA is an effective, efficient, and well-tolerated method to administer surfactant in the right patient. Second, we recognize that the use of atropine varied between the two groups. Per workshop recommendations, atropine was given prior to SAD placement in the SALSA group. Atropine administration prior to laryngoscopy was not, however, routine in the Al Bashir NICU, therefore, infants who received surfactant via InSurE did not receive atropine. A recent study has, however, shown that significant bradycardia is not prevented by the use of atropine [30]. We no longer premedicate with atropine and doubt that there was any beneficial effect in the SALSA group.

A limitation of more widespread use in our hospital was the difficulty in obtaining more SADs. Our initial supply was exhausted just as the global COVID-19 pandemic started. Despite the SAD being an effective way to deliver surfactant, manufacturers have not yet rectified the supply chain issues and there currently is not a size available which would allow them to be used in smaller neonates. Increased availability of the devices along with widespread education of their use may play an important role in decreasing worldwide infant mortality rates.

## 5. Conclusions

We have shown that the SALSA technique can be successfully implemented into the daily practice of a busy NICU in a middle-income country. This represents the first report of SALSA in this setting. We have also demonstrated the ease in which this was done and the potential benefit to our patient population. This project could be easily replicated in NICUs where an effective and efficient method of minimally invasive surfactant administration is desired.

## Figures and Tables

**Table 1:** Characteristics of the Study Cohorts †.

Sample Characteristic	SALSA (*n* = 110)	InSurE (*n* = 110)	*p*-Value
Birth weight (kg)	1.8 (0.5)	1.8 (0.5)	0.39
Gestational age (weeks)	32.5 (2.3)	32.6 (2.8)	0.69
Sex (Female)	53 (48.2)	64 (58.1)	0.18
RSS at time of surfactant administration	2.7 (0.4)	2.9 (0.4)	0.024
HR during procedure	113 (10)	105 (12)	<0.0001
Premedication with atropine	108 (98.2)	0 (0)	<0.0001

† For sex and premedication with atropine n (%) provided with Fisher’s exact *p*-value; for all other variables mean (SD) with Pitman’s permutation test *p*-value.

**Table 2:** Study Outcome †.

Outcome	SALSA (*n* = 110)	InSurE (*n* = 110)	*p*-Value
Required second dose of surfactant	6 (5.5)	17 (15.5)	0.026
Required intubation for mechanical ventilation	10 (9.1)	23 (20.9)	0.022
Treated for pneumothorax	2 (1.8)	9 (8.2)	0.059
Duration of procedure	92.9 (10.5)	111.3 (13.2)	<0.0001
RSS			
4 h	2.0 (0.5)	2.2 (0.6)	0.1919
8 h	1.5 (0.5)	1.9 (0.6)	0.0004

† For duration of procedure mean (SD) provided with Pitman’s permutation test *p*-value; RSS, mean (SD) was provided with *p*-values based on a multivariate linear model. For all other variables n (%) provided with Fisher’s exact *p*-value.

## Data Availability

The data collected for this project and the dataset used for statistical analysis can be found at: https://docs.google.com/spreadsheets/d/12IpIhre8AF34_EM0f_udrkTM3idD2_ST/edit?usp=sharing&ouid=103530624047870281808&rtpof=true&sd=true (accessed on 25 June 2022).

## References

[1] McPherson C., Wambach J.A. (2018). Prevention and Treatment of Respiratory Distress Syndrome in Preterm Neonates. Neonatal. Netw..

[2] Avery M.E., Mead J. (1959). Surface properties in relation to atelectasis and hyaline membrane disease. AMA J. Dis. Child..

[3] Nkadi P.O., Merritt T.A., Pillers D.A. (2009). An overview of pulmonary surfactant in the neonate: Genetics, metabolism, and the role of surfactant in health and disease. Mol. Genet. Metab..

[4] Parkash A., Haider N., Khoso Z.A., Shaikh A.S. (2015). Frequency, causes and outcome of neonates with respiratory distress admitted to Neonatal Intensive Care Unit, National Institute of Child Health, Karachi. J. Pak. Med. Assoc..

[5] Khader Y.S., Alyahya M., Batieha A. (2019). Perinatal and neonatal mortality in Jordan. Handbook of Healthcare in the Arab World.

[6] Halliday H.L. (2005). History of surfactant from 1980. Neonatology.

[7] Finan E., Bismilla Z., Campbell C., Leblanc V., Jefferies A., Whyte H.E. (2012). Improved procedural performance following a simulation training session may not be transferable to the clinical environment. J. Perinatol..

[8] Ehrlich P.F., Seidman P.S., Atallah O., Haque A., Helmkamp J. (2004). Endotracheal intubations in rural pediatric trauma patients. J. Pediatr. Surg..

[9] Foglia E.E., Ades A., Sawyer T., Glass K.M., Singh N., Jung P., Quek B.H., Johnston L.C., Barry J., Zenge J. (2019). Neonatal intubation practice and outcomes: An international registry study. Pediatrics.

[10] Hatch L.D., Grubb P.H., Lea A.S., Walsh W.F., Markham M.H., Whitney G.M., Slaughter J.C., Stark A.R., Ely E.W. (2016). Endotracheal intubation in neonates: A prospective study of adverse safety events in 162 infants. J. Pediatr..

[11] Khatami S.F., Parvaresh P., Behjati S. (2011). Common complications of endotracheal intubation in newborns. Iran. J. Neonatol. IJN.

[12] Erdeve Ö., Okulu E., Roberts K.D., Guthrie S.O., Fort P., Kanmaz Kutman H.G., Dargaville P.A. (2021). Alternative methods of surfactant administration in preterm infants with respiratory distress syndrome: State of the art. Turk. Arch. Pediatr..

[13] Verder H., Robertson B., Greisen G., Ebbesen F., Albertsen P., Lundstrøm K., Jacobsen T. (1994). Surfactant therapy and nasal continuous positive airway pressure for newborns with respiratory distress syndrome. Danish-Swedish Multicenter Study Group. N. Engl. J. Med..

[14] Jobe A.H. (2016). Mechanisms of Lung Injury and Bronchopulmonary Dysplasia. Am. J. Perinatol..

[15] De Bisschop B., Derriks M.F., Cools F. (2020). Early predictors for INtubation-SURfactant-Extubation failure in preterm infants with neonatal respiratory distress syndrome: A systematic review. Neonatology.

[16] Kribs A., Pillekamp F., Hu€nseler C., Vierzig A., Roth B. (2007). Early administration of surfactant in spontaneous breathing with nCPAP: Feasibility and outcome in extremely premature infants (postmenstrual age </=27 weeks). Paediatr. Anaesth..

[17] Dargaville P.A., Aiyappan A., Cornelius A., Williams C., De Paoli A.G. (2011). Preliminary evaluation of a new technique of minimally invasive surfactant therapy. Arch. Dis. Child. Fetal Neonatal Ed..

[18] Guthrie S.O., Fort P., Roberts K.D. (2021). Surfactant administration through laryngeal or supraglottic airways. Neoreviews.

[19] Bansal S.C., Caoci S., Dempsey E., Trevisanuto D., Roehr C.C. (2018). The laryngeal mask airway and its use in neonatal resuscitation: A critical review of where we are in 2017/2018. Neonatology.

[20] Pejovic N.J., Trevisanuto D., Lubulwa C., Myrnerts Höök S., Cavallin F., Byamugisha J., Nankunda J., Tylleskär T. (2018). Neonatal resuscitation using a laryngeal mask airway: A randomised trial in Uganda. Arch. Dis. Child..

[21] Trevisanuto D., Micaglio M., Ferrarese P., Zanardo V. (2004). The laryngeal mask airway: Potential applications in neonates. Arch. Dis. Child. Fetal Neonatal Ed..

[22] Wanous A.A., Wey A., Rudser K.D., Roberts K.D. (2017). Feasibility of laryngeal mask airway device placement in neonates. Neonatology.

[23] Roberts K.D., Brown R., Lampland A.L., Leone T.A., Rudser K.D., Finer N.N., Rich W.D., Merritt T.A., Czynski A.J., Kessel J.M. (2018). Laryngeal mask airway for surfactant administration in neonates: A randomized, controlled trial. J. Pediatr..

[24] Sadeghnia A., Tanhaei M., Mohammadizadeh M., Nemati M. (2014). A comparison of surfactant administration through i-gel and ET-tube in the treatment of respiratory distress syndrome in newborns weighing more than 2000 grams. Adv. Biomed. Res..

[25] Amini E., Sheikh M., Shariat M., Dalili H., Azadi N., Nourollahi S. (2019). Surfactant administration in preterm neonates using laryngeal mask airway: A randomized clinical trial. Acta Med. Iran..

[26] Pinheiro J., Santana-Rivas Q., Pezzano C. (2016). Randomized trial of laryngeal mask airway versus endotracheal intubation for surfactant delivery. J. Perinatol..

[27] Zapata H.A., Fort P., Roberts K.D., Kaluarachchi D.C., Guthrie S.O. (2022). Surfactant administration through laryngeal or supraglottic airways (SALSA): A viable method for low-income and middle-income countries. Front. Pediatr..

[28] (2019). The World Bank. https://data.worldbank.org/indicator/SH.DYN.NMRT?locations=PE.

[29] Iyer N.P., Mhanna M.J. (2013). Non-invasively derived respiratory severity score and oxygenation index in ventilated newborn infants. Pediatr. Pulmonol..

[30] Jehier A., El-Naggar W., Baier J., Hatfield T.R., Narvey M.R. Atropine versus placebo for neonatal intubation: A randomized clinical trial. Proceedings of the Pediatric Academic Societies Meeting.

